# Of neoliberalism and global health: human capital, market failure and sin/social taxes

**DOI:** 10.1080/09581596.2016.1196288

**Published:** 2016-06-13

**Authors:** David Reubi

**Affiliations:** ^a^ Department of Global Health and Social Medicine, King’s College London, London, UK

**Keywords:** Global health, neoliberalism, human capital, market failure, taxation, Foucault

## Abstract

This article tells a different but equally important story about neoliberalism and global health than the narrative on structural adjustment policies usually found in the literature. Rather than focus on macroeconomic structural adjustment policies, this story draws our attention to microeconomic taxation policies on tobacco, alcohol and sugar now widely recognised as the best strategy to control the global non-communicable disease epidemic. Structural adjustment policies are the product of the shift from statist to market-based development models, which was brought about by neoliberal thinkers like Peter Blau and Deepak Lal. In contrast, taxation policies are the result of a different epistemological rupture in international development: the move from economies and physical capital to people and human capital, advocated by Gary Becker and others. This move was part of wider change, which saw Chicago School economists, under the influence of rational choice theory, redefine the object of their discipline, from the study of markets to individual choices. It was this concern with people and their choices that made it possible for Becker and others to identify the importance of price for the demand for tobacco, alcohol and sugar. The same concern also made it easier for them to recognise that there were inefficiencies in the tobacco, alcohol and sugar markets that required government intervention. This story, I suggest, shows that structural adjustment policies and pro-market ideology do not exhaust the relationship between neoliberalism and global health and should not monopolise how we, as political and social scientists, conceive it.

People are the center of what we should be thinking about when we think about economies, when we think about development. That’s a liberating point of view. (Gary Becker, Chicago School Economist and Neoliberal Thinker)

As Bell and Green (2016) remark in their recent editorial, the political and social science literature on health and biomedicine has been traversed by an epidemic of neoliberalism of late. Indeed, perusing this literature, one is left with the impression that everything, from biomedical research to patienthood, has become ‘neoliberal’. The field of global health and development is no exception. Political and social scientists have repeatedly bemoaned how the market-based ideology of neoliberalism has negatively impacted the organisation and administration of medical care and public health in developing countries, leading to higher morbidity and mortality rates (e.g. Keshavjee, 2014; Rowden, 2009; Thomas & Weber, 2004). Though few of these scholars spend much time articulating what they mean by neoliberalism, they usually refer to the structural adjustment policies that have dominated international development from the early 1980s, when they became a standard lending modality of general budgetary support loans from the International Monetary Fund (IMF) and World Bank (Mirowski & Plehwe, 2009). These macroeconomic policies include currency devaluation, reducing public deficits, abolishing trade barriers, deregulating key industries and privatising state-owned enterprises. They grew out of the critique, articulated by neoliberal thinkers like Peter Blau, Herbert Frankel and Deepak Lal, of the statist model at the heart of post-war development economics (Collier, 2011). For them, economic development could not occur through the state, which they deemed incompetent and wasteful, but only through trade and entrepreneurship. Developing countries, they advised, had to roll back the state and open up economic activity to market forces.

Drawing on Foucault’s (2008) and others’ (e.g. Collier, 2011; Mirowski & Plehwe, 2009) work on neoliberal thought and the government of life, I tell here a very different, yet equally important story about neoliberalism and global health. Unlike the well-rehearsed narrative on structural adjustment policies, this story focuses on what are often referred to as sin taxes – taxes on tobacco, alcohol and sugar. Specifically, I examine how a group of economists centred around the Chicago School professor and prominent neoliberal thinker Gary Becker played an important role in articulating and promoting these taxes as a strategy against the global non-communicable disease (NCD) epidemic. I also analyse some of the concepts and concerns – human capital, rational choice, market failure – that informed these economists’ work on sin taxes. I conclude by suggesting that this new, different story demonstrates that narratives about structural adjustment policies and pro-market ideology do not exhaust the relationship between neoliberalism and global health and should not monopolise how we, as political and social scientists, conceive it.

## Sin taxes and the global NCD epidemic

Today, taxing tobacco, alcohol and sugar is viewed as one of the best tools to combat the global NCD epidemic (WHO, 2010; World Bank, 2011). As I discussed elsewhere (Reubi, 2013), taxes on tobacco products were the first type of sin tax to be recognised as an effective global health strategy. During the 1980s, North American and European economists began to identify price as a critical factor in reducing demand for tobacco products, suggesting that tax increases were one of the most powerful measures to combat smoking. They also calculated that taxation was particularly effective with young adults and lower socio-economic groups and showed that a tax rise did not only improve a country’s health but also its finances, as the tax rise compensated the decrease in cigarette consumption. First picked up by activists and policymakers in the West, these findings were later disseminated to developing countries through the World Bank’s (1999) report *Curbing the Epidemic*, the WHO’s 2003 Framework Convention on Tobacco Control and global health initiatives like the Bloomberg Initiative to Reduce Tobacco Use funded by the Bloomberg and Gates foundations. More recently, building on the success of tobacco taxes and new research by economists on the role of price on the demand for alcoholic and sugar-sweetened beverages, public health experts have begun to argue for the taxation of alcohol and sugar as well (Blecher, 2015). The WHO and World Bank now recommend taxing these products to fight the global NCD epidemic and a growing number of developing countries have started experimenting with this new sort of interventions (PAHO, 2015; WHO, 2010; World Bank, 2011).

While many economists have worked on sin taxes, a small group of them including Gary Becker, Mike Grossman and Frank Chaloupka has been particularly influential in converting them into a recognised global health strategy. Becker is certainly the most well known among them. A professor at Chicago from the late 1960s onwards, Becker was also, together with his former colleague Milton Friedman, a member of the neoliberal think-tank The Mont Pèlerin Society (Mirowski & Plehwe, 2009). Together with George Stigler, he was critical in articulating Chicago’s microeconomic tradition, for which he was awarded the 1992 Nobel Prize. Although Becker did not work specifically on sin taxes, he discussed them in his research on addiction and the liberalisation of drug policies (Becker, 1997). A student of Becker, Grossman is renowned for using the Chicago microeconomic approach to study health-related behaviours. It is as part of this work that he and some colleagues demonstrated the strong inverse correlation between the price of and the demand for cigarettes, forever changing the field of tobacco control (Grossman, 1985; Lewit, Coate, & Grossman, 1981). Chaloupka was Grossman’s student and then colleague, working with him on the relation between price and demand in relation to tobacco and alcohol and also contributing to Becker’s research on addiction (Chaloupka, 1990; Chaloupka & Grossman, 1996). Now an international expert on sin taxes, Chaloupka was critical in promoting tobacco taxation as a public health tool across developing countries, co-authoring *Curbing the Epidemic* and helping set-up taxation systems in Asia and Africa as a part of efforts funded by the Rockefeller, Gates and Bloomberg foundations (Reubi, 2013). More recently, he and his colleagues have also advocated for taxation on alcoholic and sugar-sweetened beverages to combat the global NCD epidemic, supporting, for example, Bloomberg Philanthropies’ sugar tax project in Mexico (Blecher, 2015; Chaloupka, 2012).

## Human capital, rational choice and social taxes

These economists’ influential work on sin taxes grew out of the post-1960s Chicago microeconomic tradition. For the first generations of Chicago economists, from Frank Knight to Milton Friedman, economics was the study of the ‘social organisation of economic activity’ and, in particular, ‘markets as coordinating devices’ (Medema, 2011, p. 153). This, like historian and economist Steven Medema (2011) has showed, changed after the 1960s following the arrivals of Stigler and Becker at Chicago. For this new generation influenced by rational choice theory, economics became the study of human behaviour and, specifically, rational individual choices under conditions of scarcity. As Foucault (2008) has argued, this shift had two consequences for economics. First, by ‘putting people at the centre of the economy’ (Becker, Ewald, & Harcourt, 2011, p. 17), it made it possible to analyse how individual decisions had implications at the macro level, thus ‘exten[ding] economic analysis within its own domain’ (Foucault, 2008, p. 215). Second, by making any human behaviour a potential object of study, it encouraged an imperialistic agenda among economists who, following Becker’s lead, increasingly applied their methods to traditionally non-economic domains. Becker (1964) and others’ work on human capital was particularly important in realising this shift from economic system to rational choice and articulating Chicago’s microeconomic tradition. In contrast to traditional economics, which narrowly associated economic development and growth with physical capital like roads and factories, they argued that development and growth were equally, if not more, related to human capital like education and health (Foucault, 2008). For human capital theorists like Becker, individuals had a set of bodily and intellectual capacities that were both a capital and a source of income. These individuals, they thought, could decide to augment their capital by investing in themselves through education and healthcare. The decision to invest, they also assumed, was one that individuals – understood as rational beings seeking to maximise their welfare and ‘entrepreneurs of themselves’ (Foucault, 2008, p. 226) – took by weighing the price to pay (in time and money) and potential gains (in earnings and wellbeing). For the likes of Becker then, the economist’s task was to calculate (by drawing on survey data, theoretical models and econometric tools) the demand for investment in human capital in different social groups and point out to policymakers the under-investments in need of remedy (Becker et al., 2011).

It was not long before the Chicago microeconomic approach together with human capital and rational choice theories were applied to health. Examples abound, from Selma Mushkin’s (1962) *Health as an Investment* to the World Bank’s (1993) *Investing in Health*. Grossman was one of the leaders in this field, both in his own research and as the director of the National Bureau of Economic Research’s Health Economics Programme. In the 1970s, he adapted Becker’s human capital model to study why individuals choose to invest in good health, showing how factors like education and age affected demand by altering the shadow price of health (Grossman, 1972). And, later on, together with Chaloupka and others, he explored why people choose to smoke and drink (e.g. Chaloupka & Grossman, 1996; Grossman, 1985). This research drew on Becker’s rational addiction model (Becker & Murphy, 1988) and showed that the consumption of addictive goods like tobacco and alcohol was a cogent choice made by utility-maximising individuals on the basis of factors like income, pleasure and price (e.g. Becker, Grossman, & Murphy, 1994; Chaloupka, 1990). On the one hand, Grossman and others’ microeconomic approach to health-related behaviours and, especially, the lifestyles associated with chronic diseases was not surprising. Indeed, this was a period when, responding to the political concerns about rising health expenditures in North America and Europe, there was a growing interest in health among economists (Reubi, 2013). And, with chronic diseases being the prime health issue in the West at that time, it was logical that most health economists worked on these diseases and associated risk factors (Armstrong, 2014). On the other hand, it is important to stress that the approach used by the likes of Grossman was very different from the approaches adopted by other economists working on health. The latter comprised industrial organisation economists interested in how the healthcare industry was structured and public policy economists calculating the cost of diseases to the economy (Reubi, 2013). In contrast, Grossman and his like were concerned with health-related individual behaviours and the factors influencing these. It was this concern with people and their choices, rather than with the organisation of healthcare or the disease burden for the economy, that made it possible for them to identify the inverse correlation between price and demand in relation to tobacco, alcohol and sugar.

For Becker, Grossman, Chaloupka and their colleagues, sin taxes were desirable not only because they decreased demand for tobacco, alcohol and sugar and thus saved lives, but also because they remedied existing failures in the markets for these products. Specifically, they argued that these markets were characterised by two ‘market failures’ (Jha & Chaloupka, 2000, p. 155). The first was an ‘information failure’ (Jha & Chaloupka, 2000, p. 153). Economic theory assumed that in a functional market, consumers are informed about all the risks and benefits of the products they purchase. This, Becker and his colleagues suggested, was not the case in the tobacco, alcohol and sugar markets: while some consumers had no knowledge about the health risks associated with these products, others knew, but failed to appreciate their scale or to apply them to their own situation (Jha & Chaloupka, 2000, pp. 156–159). The second failure related to what Becker (1997, p. 150) termed the ‘social cost’ of smoking, drinking and eating sugar. Economists generally presume that within a free, competitive market consumers bear all the costs associated with their choices. Again, Becker and his colleagues argued that this assumption did not hold for the tobacco, alcohol and sugar markets, where many costs were not borne by consumers but, instead, imposed on society. These costs included the health impact of passive smoking, accidents due to drink driving and the expenses for tobacco-, alcohol- and sugar-related morbidity covered by social insurance schemes (Becker, 1997; Jha & Chaloupka, 2000). For Becker and his colleagues, these market failures resulted in ‘economic inefficiencies’ that had to be corrected through ‘government interventions’ (Jha & Chaloupka, 2000, pp. 153, 168). Sin taxes, they argued, were the most effective measure that governments could use to do so. By increasing the price of tobacco, alcohol and sugar, these taxes – which Becker (1997, p. 150) called ‘social taxes’ – reduced the demand for such products; a demand which was previously deemed to be artificially inflated because of consumers’ failure to understand the risks and bear the costs associated with these products. It is important to note here that this position defended by Becker and his colleagues that the tobacco, alcohol and sugar markets were flawed and had to be corrected through increased taxation drew fierce opposition from some neoliberal thinkers, for whom any government intervention in the market was anathema. These thinkers included followers of Ludwig von Mises and the Austrian School as well as figures like Deepak Lal, who had spearheaded the critique of the post-war state-centred development model (Mamudu, Hammond, & Glantz, 2008; Thornton, 2005). This opposition should probably not come as a surprise, if one remembers the multiple and often conflicting intellectual traditions that make up the neoliberal movement (Collier, 2011; Mirowski & Plehwe, 2009).

## Conclusion

This article laid out a different but equally important story about neoliberalism and global health than the well-rehearsed narrative on structural adjustment policies usually found in the literature. Rather than focus on macroeconomic structural adjustment policies, this story draws our attention to the microeconomic taxation policies now widely recognised as the best strategy to control the NCD epidemic. As it is well-known, structural adjustment policies are the product of the shift from statist to market-based development models, which was brought about by a network of neoliberal thinkers like Blau, Frankel and Lal. In contrast, I showed that taxation policies are the result of a different epistemological rupture in international development: the move from economies and physical capital to people and human capital, advocated by Becker and others. This move was a part of a wider change, which saw Chicago School economists, under the influence of rational choice theory, redefine the object of their discipline, from the study of markets to individual choices. As I illustrated, it was this concern with people and their choices that made it possible for Becker and his followers like Grossman and Chaloupka to identify the importance of price for the demand for tobacco, alcohol and sugar. The same concern also made it easier for them to recognise that there were inefficiencies (in the form of information failures and external social costs) in the tobacco, alcohol and sugar markets that required government intervention, something other neoliberal thinkers like Lal openly criticised. Beyond the specificities of the case, the story outlined here also offers some more general insights on how one can fruitfully study the relationship between neoliberalism and (global) health. To start with, this story reminds us that the neoliberal movement is inherently plural and divided, with a multiplicity of schools promoting their own and frequently conflicting traditions and concerns. Furthermore, this story also suggests that it might be more productive to follow existing neoliberal networks and empirically study their sometimes counter-intuitive intellectual agendas rather than start from a broad, conventional understanding of what neoliberalism is and then identify as ‘neoliberal’ anything that somewhat fits this predetermined definition. Finally, and more provocatively perhaps, the story told here asks whether, following Foucault, it might be more fertile to abandon the negative apriori many of us in the political and social sciences hold vis-à-vis all things neoliberal and adopt instead a ‘general *penchant* [or] favour for neoliberalism’ when studying its relationship with health (Becker et al., 2011, p. 11).

## Disclosure statement

No potential conflict of interest was reported by the author.
